# Background Adjusted Alignment-Free Dissimilarity Measures Improve the Detection of Horizontal Gene Transfer

**DOI:** 10.3389/fmicb.2018.00711

**Published:** 2018-04-16

**Authors:** Kujin Tang, Yang Young Lu, Fengzhu Sun

**Affiliations:** ^1^Molecular and Computational Biology Program, Department of Biological Sciences, University of Southern California, Los Angeles, CA, United States; ^2^Centre for Computational Systems Biology, School of Mathematical Sciences, Fudan University, Shanghai, China

**Keywords:** horizontal gene transfer, genomic island, alignment-free, d^*^_2_, *CVTree*, kmer

## Abstract

Horizontal gene transfer (HGT) plays an important role in the evolution of microbial organisms including bacteria. Alignment-free methods based on single genome compositional information have been used to detect HGT. Currently, Manhattan and Euclidean distances based on tetranucleotide frequencies are the most commonly used alignment-free dissimilarity measures to detect HGT. By testing on simulated bacterial sequences and real data sets with known horizontal transferred genomic regions, we found that more advanced alignment-free dissimilarity measures such as *CVTree* and $d_{2}^{*}$ that take into account the background Markov sequences can solve HGT detection problems with significantly improved performance. We also studied the influence of different factors such as evolutionary distance between host and donor sequences, size of sliding window, and host genome composition on the performances of alignment-free methods to detect HGT. Our study showed that alignment-free methods can predict HGT accurately when host and donor genomes are in different order levels. Among all methods, *CVTree* with word length of 3, $d_{2}^{*}$ with word length 3, Markov order 1 and $d_{2}^{*}$ with word length 4, Markov order 1 outperform others in terms of their highest *F*_1_-score and their robustness under the influence of different factors.

## Introduction

As opposed to vertical transmission in which DNA is transferred from parent to offspring, horizontal gene transfer (HGT) or lateral gene transfer (LGT) is defined as the movement of genetic material between organisms that are not in a parent-offspring relationship. HGT plays an important role in bacterial evolution as it is the primary reason underlying the adaptation of bacteria such as metabolic adaptation (Pál et al., 2005) and antibiotic resistance (Gyles and Boerlin, 2014). Both alignment-based and alignment-free methods have been used to infer horizontal gene transfer (Karlin and Burge, 1995; Karlin, 2001; Tsirigos and Rigoutsos, 2005; Becq et al., 2010; Langille et al., 2010; Ravenhall et al., 2015; Cong et al., 2016a,b, 2017; Lu and Leong, 2016). Alignment-based, or phylogenetic methods, are often considered as the gold standard (Keeling and Palmer, 2008) for HGT detection because of their explicit model. Such methods detect horizontal gene transfer by integrating information from multiple organisms to find genes whose phylogenetic relationships among multiple organisms differ significantly from that of other genes (Ravenhall et al., 2015; Lu and Leong, 2016). Despite their extensive applications in horizontal gene transfer detection, finding topological incongruences is time-consuming, uses large memory, and requires that genomes of interest have to be annotated and their phylogenetic relationships are known. In addition, alignment-based methods can only be applied to gene or protein sequences and thus limit their ability to detect horizontal transfer in non-coding regions.

On the other hand, alignment-free, also called compositional parametric, methods detect horizontal gene transfer based on the detection of regions in a genome with atypical word pattern (kmer, ktuple, kgram, etc.) composition. These methods are based on the observation that different microbial species have their own genomic word pattern signatures (Karlin and Burge, 1995) so that sequences transferred from donor genome are likely to have different composition signatures from that of the host genome. DNA acquired via horizontal gene transfer will, over time, acquire the composition signatures of the host genome through a process called amelioration (Lawrence and Ochman, 1997). Recently, Cong et al. (2016a,b, 2017) introduced TF-IDF as a scalable alignment-free approach for HGT detection by combining multiple genomes and kmer occurrences. However, this method assumes that the donor genome is in the group of genomes under study and requires phylogenetic relationships among these genomes. More widely used alignment-free methods apply a sliding window to scan a single genome and calculate the distance between the composition of each window and the whole genome. Consecutive windows with distance from the whole genome higher than a threshold are inferred as HGT. The performances of alignment-free methods depend largely on the choice of genomic signatures. Commonly-used genomic signatures include, but are not limited to GC content (Karlin, 2001), codon usage (Karlin, 2001) and oligonucleotide (kmer) frequencies (Tsirigos and Rigoutsos, 2005). Becq et al. (2010) reviewed alignment-free methods on horizontal gene transfer detection and showed that kmer-based methods with a 5 kbp sliding window outperformed other alignment-free methods based on features such as GC content (Karlin, 2001), codon usage (Karlin, 2001) and dinucleotides (Karlin and Burge, 1995). However, they only tested Euclidean distance with kmer length 4 as genomic signature (Dufraigne et al., 2005) for kmer-based methods. In fact, the performances of kmer-based methods can vary largely depending on the choice of the value of *k* and dissimilarity measures between kmer vectors.

For kmer-based methods, Manhattan and Euclidean distances between the kmer frequency vector of a genomic region and that of the whole genome are the most frequently used measures for detecting HGTs because of their simplicity. For example, Dufraigne analyzed HGT regions of 22 genomes by using Euclidean distance with kmer length 4 (Dufraigne et al., 2005). In addition, they compared the genomic signatures of HGT regions with 12,000 species from GeneBank by Euclidean distance to find their potential donors. Rajan et al. used Manhattan distance with k-mer length 5 to detect HGT in 50 diverse bacterial genomes (Rajan et al., 2007). Tsirigos and Rigoutsos (2005) proposed to use relative kmer frequencies defined by the absolute kmer frequency over the expected frequency under the independent identically distributed (IID) model for HGT detection. They also investigated a few dissimilarity measures between the relative frequencies of a genomic region and the whole genome including correlation, covariance, Manhattan distance, Mahalanobis distance, and Kullback–Leibler (KL) distance for HTG detection. They showed that kmers of length 6–8 with covariance dissimilarity perform the best under their simulated situations. Several review papers on the use of kmers for the detection of HGT are available (Langille et al., 2010; Ravenhall et al., 2015; Lu and Leong, 2016). As in most studies of HGT, we concentrate on the use of kmers for HGT detection by using a single genome in this paper.

Recently, several new dissimilarity measures for sequence comparison based on kmer frequency vectors have been developed including *CVTree* (Qi et al., 2004), $d_{2}^{*}$ and $d_{2}^{s}$ (Reinert et al., 2009; Song et al., 2013; Lu et al., 2017). They have been shown to out-perform commonly used measures such as Manhattan and Euclidean distances for solving different problems including evolutionary distance estimation (Ren et al., 2016), virus-host interaction prediction (Ahlgren et al., 2017), and metagenome and metatranscriptome comparison (Jiang et al., 2012; Liao et al., 2016). However, these dissimilarity measures have not been used for HGT detection. It is important to know whether these new dissimilarity measures have better performance than available methods for detecting horizontal gene transfers. In addition, it is important to study the influence of evolutionary distance between host and donor genomes, sliding window size, and different host genome compositions on the performance of kmer-based alignment-free methods on HGT detection. In this study, we have addressed all these issues.

## Materials and methods

### Artificial genome simulation

We chose *Escherichia coli* K12 (*E. coli*) as the host genome and *Bacillus subtilis* 168 (*B. subtilis*), *Haemophilus influenzae* Rd KW20 (*H. influenzae*), *Helicobacter pylori* 26695 (*H. pylori*), *Mycobacterium tuberculosis* H37RV (*M. tuberculosis*), and *Streptococcus pneumoniae* R6 (*S. pneumoniae*) as donor genomes. Each time, we picked a fragment randomly from the donor genome with length uniformly chosen from 8kbp to 40kbp and inserted it into a random position uniformly along the *E. coli* K12 genome until the simulated HGT consists of up to 10% of the artificial genome, since the HGT proportions in most bacteria genomes range from 2 to 15% (Garcia-Vallvé et al., 2000). We named the simulated genome as “*E. coli*_artificial.” To make our results more reliable, we did 10 simulations. Table S1 in the Supplementary Material shows the detailed composition of one of these 10 simulated genomes.

One of the challenges for evaluating HGT detection methods is the lack of a benchmark data. The host genome may contain genes historically transferred from other genomes, but they are not part of the simulated transferred regions. If a HGT detection method predicts such a gene as a HGT, although the prediction is correct, the prediction will be reported as a false positive since the gene is not transferred through the simulation. Therefore, the reported false positive rate maybe higher than the true false positive rate. On the other hand, such a problem is common to all the HGT detection methods and their relative performances are still valid. Therefore, we can still use artificial genomes to compare the relative performance of different methods.

### Distance/dissimilarity measures between genomic sequences

Given two genomic sequences *i* and *j* and a given word length *k*, we first count the number of occurrences of all kmers in sequence *i* and sequence *j*, respectively. The full set of kmers of length *k* is defined as $A^{k}$ where A=(A,T,C,G) for nucleotide sequences. For a given kmer *w*, its occurrences in *i* is defined as $N_{w}^{\left(i\right)}$ and the frequency or the relative abundance of this kmer is defined as $f_{w}^{\left(i\right)}=\frac{N_{w}^{\left(i\right)}}{\underset{w}{\sum }N_{w}^{\left(i\right)}}$.

Some dissimilarity measures such as $d_{2}^{*}$ and $d_{2}^{S}$ need an *m*-th order Markov model for the background sequence. The expected number of occurrences of word *w*, $𝔼N_{w}^{\left(i\right)}$, can be calculated from the stationary probability of the first *m*-mer *w*[1 : *m*] and the transition probabilities from the *n*-th *m*-mer *w*[*n* : *n* + *m* − 1] to the (*n* + *m*)-th nucleotide *w*[*n* + *m*]:

$$𝔼N_{w}^{\left(i\right)}=(L^{\left(i\right)}-k+1)\mu (w[1:m])\prod_{n=1}^{k-m}\pi (w[n:n+m-1],w[n+m])$$

where *L*^(*i*)^ is the length of sequence *i*, μ is the stationary probability and π is the transition probability that can be estimated from the sequence data. The difference between the occurrences of kmer *w* and its expected occurrences is defined as ${Ñ}_{w}^{\left(i\right)}=N_{w}^{\left(i\right)}-𝔼N_{w}^{\left(i\right)}$.

#### Manhattan

The Manhattan distance (Ma) is defined as:

$$Ma=\sum_{w\in A^{k}}\left|f_{w}^{\left(i\right)}-f_{w}^{\left(j\right)}\right|$$

#### Euclidean

The Euclidean distance (Eu) is defined as:

$$Eu=\sqrt{\sum_{w\in A^{k}}|f_{w}^{\left(i\right)}-f_{w}^{\left(j\right)}{|}^{2}}$$

#### *d*_2_ (Torney et al., 1990)

The *d*_2_ distance is defined as:

$$d_{2}=\frac{1}{2}\left(1-\frac{\sum_{w\in A^{k}}f_{w}^{\left(i\right)}f_{w}^{\left(j\right)}}{\sqrt{\sum_{w\in A^{k}}{\left(f_{w}^{\left(i\right)}\right)}^{2}}\sqrt{\sum_{w\in A^{k}}{\left(f_{w}^{\left(j\right)}\right)}^{2}}}\right)$$

#### *CVTree* (Qi et al., 2004)

The *CVTree* dissimilarity is defined as:

$$CVTree=\frac{1}{2}\left(1-\frac{\sum_{w\in A^{k}}{\hat{f}}_{w}^{\left(i\right)}{\hat{f}}_{w}^{\left(j\right)}}{\sqrt{\sum_{w\in A^{k}}{\left({\hat{f}}_{w}^{\left(i\right)}\right)}^{2}}\sqrt{\sum_{w\in A^{k}}{\left({\hat{f}}_{w}^{\left(j\right)}\right)}^{2}}}\right)$$

where ${\hat{f}}_{w}^{\left(i\right)}=\frac{{Ñ}_{w}^{\left(i\right)}}{𝔼N_{w}^{\left(i\right)}}$. *CVTree* calculates $𝔼N_{w}^{\left(i\right)}$ by assuming a (*k* − 2)-th order Markov chain for genomic sequences.

#### $d_{2}^{*}$ (Reinert et al., 2009)

The $d_{2}^{*}$ dissimilarity is defined as:

$$d_{2}^{*}=\frac{1}{2}\left(1-\frac{\sum_{w\in A^{k}}{\bar{f}}_{w}^{\left(i\right)}{\bar{f}}_{w}^{\left(j\right)}}{\sqrt{\sum_{w\in A^{k}}{\left({\bar{f}}_{w}^{\left(i\right)}\right)}^{2}}\sqrt{\sum_{w\in A^{k}}{\left({\bar{f}}_{w}^{\left(j\right)}\right)}^{2}}}\right)$$

where ${\bar{f}}_{w}^{\left(i\right)}=\frac{{\tilde{N}}_{w}^{\left(i\right)}}{\sqrt{𝔼N_{w}^{\left(i\right)}}}$.

#### $d_{2}^{s}$ (Reinert et al., 2009)

The $d_{2}^{s}$ dissimilarity is defined as:

$$d_{2}^{s}=\frac{1}{2}\left(1-\frac{\sum_{w\in A^{k}}{\tilde{f}}_{w}^{\left(i\right)}{\tilde{f}}_{w}^{\left(j\right)}}{\sqrt{\sum_{w\in A^{k}}{\left({\tilde{f}}_{w}^{\left(i\right)}\right)}^{2}}\sqrt{\sum_{w\in A^{k}}{\left({\tilde{f}}_{w}^{\left(j\right)}\right)}^{2}}}\right)$$

where ${\tilde{f}}_{w}^{\left(i\right)}=\frac{{\tilde{N}}_{w}^{\left(i\right)}}{{\left({\left({\tilde{N}}_{w}^{\left(i\right)}\right)}^{2}+{\left({\tilde{N}}_{w}^{\left(j\right)}\right)}^{2}\right)}^{\frac{1}{4}}}$ and ${\tilde{f}}_{w}^{\left(j\right)}=\frac{{\tilde{N}}_{w}^{\left(j\right)}}{{\left({\left({\tilde{N}}_{w}^{\left(i\right)}\right)}^{2}+{\left({\tilde{N}}_{w}^{\left(j\right)}\right)}^{2}\right)}^{\frac{1}{4}}}$.

### Distance calculation

As in most studies (Dufraigne et al., 2005; Tsirigos and Rigoutsos, 2005), we used a sliding window approach for the detection of HGT. Starting from the 5′-end of the *E. coli*_artificial genome, we divided the genome into overlapped windows of size *b* with sliding step of 500 bps. As suggested by Dufraigne et al. (2005), we first used *b* = 5 kbp. We uesd CAFE (Lu et al., 2017), an accelerated alignment-free sequence analysis tool, to calculate different dissimilarity measures between each window and the whole genome by using the different alignment-free dissimilarity measures with different kmer lengths and Markov orders as needed. For measure *d*_2_, Euclidean, and Manhattan, that do not require Markov order information, we used *k* = 3, 4, 5. For $d_{2}^{*}$ and $d_{2}^{s}$, we tested them with *k* = 3, 4, 5 and Markov order = 0, 1, 2, 3. For *CVTree* that assumes a Markov chain of order (*k* − 2), we tested it with *k* = 3, 4, 5. For all methods, a double-strand signature was used to remove strand compositional asymmetry (Karlin, 1999), which means we counted kmer occurrences in both the sequence and its reverse complementary sequence.

### Predicting HGT regions

Windows with high dissimilarity with the whole genome are more likely to be transferred from other genomes. Therefore, a window is predicted to be a HGT region if its dissimilarity with the whole genome *D* is above a certain threshold *T*. We used the same criterion as in Becq et al. (2010) to determine the threshold, that is,

$$T=Q_{3}+r(Q_{3}-Q_{1}),$$

where *Q*_1_ and *Q*_3_ are the first and third quartiles of the distribution of dissimilarity values between all the windows and the whole genome, and *r* is a parameter used to set the threshold that ranges from 0.25 to 10.00 with a step of 0.25. Therefore, for each alignment-free method with certain word length *k* and Markov order *m*, we could define 40 thresholds. Windows with distance from the whole genome above the threshold were defined as atypical windows. Overlapped atypical windows were then concatenated to form atypical regions, which were predicted as HGT regions.

### Evaluation criteria

By comparing the detected HGT and the real transferred fragments in *E. coli*_artificial, we calculated the recall (sensitivity) and precision. Recall is calculated as the length of the overlapped sequence between detected HGT and simulated HGT divided by the total length of simulated HGT fragments. Precision is calculated as the length of the overlapped sequence between detected HGT and simulated HGT divided by the total length of detected HGT. A commonly used measure that combines precision and recall is the harmonic mean of precision and recall, the traditional *F*_1_-measure or balanced *F*_1_-score, defined as

$$F_{1}=2\times \frac{\text{precision}\times \text{recall}}{\text{precision}+\text{recall}}.$$

Given an *E. coli*_artificial genome, for each threshold, we calculated the precision, recall and the *F*_1_-score for each method. We then calculated the average precision, recall and the average *F*_1_-score for each threshold over 10 simulated genomes and plotted the precision-recall curve. We report the optimal *F*_1_-score for each dissimilarity measure.

Since most parts of the host genome are not transferred from other genomes, the receiver operating curve (ROC) showing the relationship between the false positive rate (FPR, 1 - specificity) and true positive rate (TPR, recall or sensitivity) is not optimal for comparing the different dissimilarity measures since the area under the ROC curve (AUC) and the specificity are generally very high. Therefore, we used the precision recall curve (PRC) and *F*_1_-score as our criterion for comparing the different dissimilarity measures.

### Investigating the effect of evolution relationship between the host and donor genomes and window size on the performance of different methods

In the simulated genome above, we assumed that all the donor genomes can contribute to the host genome through HGT. Since closely related genomes have similar kmer frequencies, it will be difficult to detect HGT from closely related genomes. On the other hand, if the donor genome has high evolutionary distance from the host genome, it will be relatively easy to identify HGT with any reasonable methods. Therefore, we next investigated how the evolutionary relationship between the the donor genome and the host genome affects the relative performance of the different HGT detection methods.

In our simulations, we still used *E. coli* K12 that is of the Proteobacteria phylum, Gammaproteobacteria class, Enterobacteriales order, Enterobacteriaceae family and Escherichia genus as host genome and chose 20 donor genomes having different evolutionary relationships with *E. coli*. Four of them are different species of the Escherichia genus [*Escherichia albertii* KF1 (*E. albertii*), *Escherichia fergusonii* ATCC 35469 (*E. fergusonii*), *Escherichia hermannii* NBRC 105704 (*E. hermannii*), *Escherichia vulneris* NBRC 102420 (*E. vulneris*)], four of them are in different genus of the Enterobacteriaceae family [*Enterobacter cloacae* ATCC 13047 (*E. cloacae*), *Klebsiella pneumoniae* HS11286 (*K. pneumoniae*), *Salmonella typhimurium* LT2 (*S. typhimurium*), *Shigella sonnei* 53G (*S. sonnei*)], four of them are in different families of the Enterobacteriales order [*Yersinia pestis* KIM 10+ (*Y. pestis*), *Photorhabdus luminescens* TT01 (*P. luminescens*), *Pantoea ananatis* LMG 20103 (*P. ananatis*), *Brenneria goodwinii* OBR1 (*B. goodwinii*)], four genomes are in different orders of the Gammaproteobacteria class [*Legionella pneumophila* Philadelphia 1 (*L. pneumophila*), *Pseudomonas aeruginosa* PA01 (*P. aeruginosa*), *Vibrio parahaemolyticus* RIMD 2210633 (*V. parahaemolyticus*), *Xanthomonas axonopodis* Xac29-1 (*X. axonopodis*)], and four genomes are in different classes of the Proteobacteria phylum [*Burkholderia pseudomallei* K96243 (*B. pseudomallei*), *Brucella abortus* 2308 (*B. abortus*), *Campylobacter coli* RM4661 (*C. coli*), *Acidithiobacillus ferrooxidans* ATCC 23270 (*A. ferrooxidans*)]. By transferring fragments between 8 and 40 kbp uniformly picked from these genomes into *E. coli* K12, we constructed 20 artificial genomes, each of them consists of 10% HGT from a certain single donor genome. We then detected the HGT using the different alignment-free methods and compared them using the same criteria as above.

In order to study the effect of window length, we continued to use the 20 artificial genomes generated above. Instead of using 5 kbp as the length of sliding window, we changed the window size to 3 and 8 kbp, respectively. Finally, we used the *F*_1_-score to evaluate the different methods.

To see if our results are consistent for different host genomes, we changed the host genome from *E. coli* to *B. abortus* and *K. pneumoniae*, respectively. Then we did the same analyses as for *E. coli*.

### Investigation of HGT within 118 genomes and *E. faecalis* V583

To evaluate the performances of alignment-free methods on HGT detection over real data, we used a data set constructed in Langille et al. (2008). In this study, the authors selected 118 genomes from 117 different strains and used a comparative genomics approach to detect genomic islands resulted from horizontal gene transfer. This benchmark data was constructed using alignments and did not use nucleotide composition information. Therefore, the data set can be used to evaluate different alignment-free HGT detection methods. For each genome, the authors provided positive and negative regions of HGT. As in Langille et al. (2008), we used precision, recall and overall accuracy to evaluate performances of alignment-free methods on HGT prediction over these 118 chromosomes, where the accuracy is calculated by the fraction of true positives and true negatives over all the predictions. In addition, we also used the optimal *F*_1_-score and the precision-recall curve to compare the different methods.

We also used the different methods to identify HGT regions of *Enterococcus faecalis* V583 (*E. faecalis*) that contains seven known genes transferred from other genomes. Since we do not know the whole set of HGT genes, we just investigated if these seven genes are ranked higher than other genes. The higher these genes are ranked by a particular method, the better performance the method is in predicting HGT.

## Results

### Background adjusted dissimilarity measures outperform non-background adjusted methods for HGT detection based on the *E. coli*_artificial genome

Table 1 shows the precision and recall yielding the highest average *F*_1_-score of the different alignment-free methods for different word size *k* and Markov order *m* when needed. The highest *F*_1_-score of 0.88 is obtained for *CVT*(4), followed by *CVT*(3), $d_{2}^{*}\left(3,1\right)$ and $d_{2}^{*}\left(4,1\right)$ (the first number in the parenthesis is the word length and the second number is the order of MC) with average *F*_1_-score at least 0.87. In comparison with background adusted dissimilarity measures, the widely-used Manhattan and Euclidean distances both have *F*_1_-score at most 0.80.

**Table 1:** Complete evaluation results for different dissimilarity measures with different word lengths *k* and Markov orders when needed.

**Method**	**Precision**	**Recall**	**Optimal *F*_1_**	**Optimal *r***
*CVT*(3)	0.77 ± 0.01	0.99 ± 0.01	0.87 ± 0.00	4.50
*CVT*(4)	0.81 ± 0.01	0.95 ± 0.02	0.88 ± 0.01	2.75
*CVT*(5)	0.70 ± 0.02	0.71 ± 0.05	0.71 ± 0.03	1.25
$d_{2}^{*}$(3, 0)	0.70 ± 0.04	0.65 ± 0.11	0.67 ± 0.08	4.75
$d_{2}^{*}$(3, 1)	0.77 ± 0.01	0.99 ± 0.00	0.87 ± 0.01	4.25
$d_{2}^{*}$(4, 0)	0.56 ± 0.01	0.96 ± 0.02	0.71 ± 0.01	2.00
$d_{2}^{*}$(4, 1)	0.77 ± 0.01	0.99 ± 0.01	0.87 ± 0.01	3.75
$d_{2}^{*}$(4, 2)	0.77 ± 0.01	0.96 ± 0.02	0.86 ± 0.01	2.25
$d_{2}^{*}$(5, 0)	0.58 ± 0.01	0.93 ± 0.03	0.71 ± 0.01	2.00
$d_{2}^{*}$(5, 1)	0.76 ± 0.01	0.98 ± 0.01	0.86 ± 0.01	3.00
$d_{2}^{*}$(5, 2)	0.82 ± 0.01	0.90 ± 0.03	0.86 ± 0.02	2.25
$d_{2}^{*}$(5, 3)	0.54 ± 0.03	0.78 ± 0.05	0.64 ± 0.03	1.00
$d_{2}^{s}$(3, 0)	0.39 ± 0.12	0.82 ± 0.21	0.49 ± 0.03	0.50
$d_{2}^{s}$(3, 1)	0.75 ± 0.01	0.99 ± 0.01	0.85 ± 0.01	2.50
$d_{2}^{s}$(4, 0)	0.54 ± 0.10	0.83 ± 0.19	0.63 ± 0.04	0.75
$d_{2}^{s}$(4, 1)	0.76 ± 0.06	0.79 ± 0.18	0.76 ± 0.09	1.00
$d_{2}^{s}$(4, 2)	0.74 ± 0.02	0.79 ± 0.13	0.76 ± 0.06	1.00
$d_{2}^{s}$(5, 0)	0.58 ± 0.02	0.80 ± 0.09	0.67 ± 0.03	1.00
$d_{2}^{s}$(5, 1)	0.74 ± 0.03	0.89 ± 0.08	0.80 ± 0.03	1.50
$d_{2}^{s}$(5, 2)	0.83 ± 0.02	0.87 ± 0.06	0.85 ± 0.03	1.50
$d_{2}^{s}$(5, 3)	0.63 ± 0.02	0.67 ± 0.08	0.65 ± 0.04	1.00
Ma(3)	0.75 ± 0.04	0.79 ± 0.12	0.76 ± 0.07	2.50
Ma(4)	0.80 ± 0.03	0.80 ± 0.12	0.80 ± 0.07	3.00
Ma(5)	0.79 ± 0.03	0.81 ± 0.12	0.80 ± 0.07	3.25
Eu(3)	0.76 ± 0.03	0.78 ± 0.13	0.76 ± 0.07	2.50
Eu(4)	0.80 ± 0.02	0.77 ± 0.12	0.79 ± 0.07	2.75
Eu(5)	0.79 ± 0.02	0.80 ± 0.12	0.79 ± 0.07	2.75
*d*_2_(3)	0.80 ± 0.04	0.76 ± 0.12	0.78 ± 0.07	5.00
*d*_2_(4)	0.77 ± 0.04	0.82 ± 0.12	0.79 ± 0.06	4.50
*d*_2_(5)	0.81 ± 0.03	0.81 ± 0.12	0.81 ± 0.07	4.50

Numbers in the brackets in the first column indicate the word length k and Markov order used by methods $d_{2}^{*}$ and $d_{2}^{S}$. For example, $d_{2}^{*}\left(3,1\right)$ means that $d_{2}^{*}$ was the dissimilarity measure with word length 3 and Markov order 1. Optimal F_1_ is the highest average F_1_-score that can be achieved by this method under a certain threshold. Optimal r for each method is the value of r, which is used to set the threshold, to achieve the optimal F_1_. Corresponding average precision and average recall for the optimal F_1_ are recorded in the second and the third columns. Standard deviations of precision, recall and optimal F_1_-score over 10 simulations are shown as superscripts. Highlighted are the top 4 F_1_-scores for the different methods.

**Figure 1:**
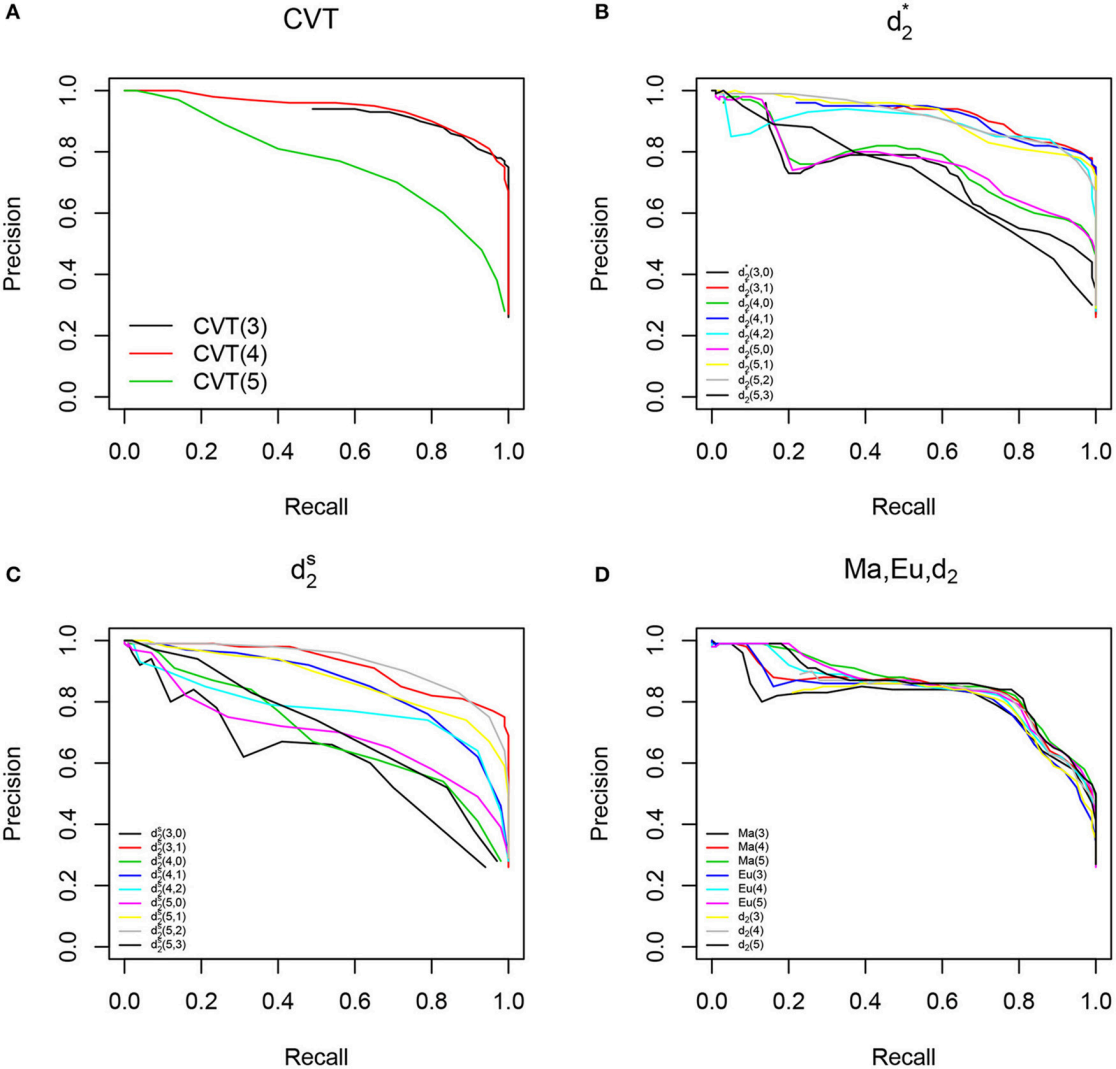
Precision-Recall Curves (PRC) for all the methods. **(A)** Shows PRC for *CVTree* with different word lengths. **(B)** Shows PRC for all the $d_{2}^{*}$ methods. **(C)** Shows PRC for all $d_{2}^{s}$ methods. **(D)** Shows PRC for Manhattan, Euclidean and *d*_2_.

In addition to comparing the different methods at the optimal *F*_1_ level, we also plotted the precision-recall curves for the different methods shown in Figure 1. Figure 1D shows that non-background adjusted methods *Ma*, *Eu* and *d*_2_ showed similar performace. Figures 1A–C show that background adjusted methods had better performance than non-background adjusted methods when *k* = 3 or *k* = 4. Among all methods, *CVT*(3), *CVT*(4), $d_{2}^{*}\left(3,1\right)$ and $d_{2}^{*}\left(4,1\right)$ had the best performace in terms of precision-recall curves. The conclusions about the relative performance of the different methods are the same based on either the *F*_1_-score or the precision-recall curves.

The better performance of the background adjusted methods (*CVTree*, $d_{2}^{*}$ and $d_{2}^{S}$) over the non-background adjusted methods (Manhattan, Euclidean, and *d*_2_) can probably be explained by the following observations. By removing the background counts of the word patterns, the signals from the most relevant kmers representative of the host genome are amplified while the contributions of irrelevant kmers are mitigated. Therefore, the background adjusted dissimilarity measures perform well in HGT detection.

Based on the performances of the different methods shown in Table 1 and Figure 1, we only present our results for the top performing methods in the rest of the paper. We chose *CVT*(3), *CVT*(4), $d_{2}^{*}\left(3,1\right)$, and $d_{2}^{*}\left(4,1\right)$ to represent background adjusted methods and Ma(5), Eu(5), and *d*_2_(5) to represent non-background adjusted methods as candidates for the following studies.

### The performance of the alignment-free methods increases with the genetic distance between the donor genome and the host genome

We next investigated the influence of evolutionary distance between the donor genome and the host genome on the performance of the different methods *CVT*(3), *CVT*(4), $d_{2}^{*}\left(3,1\right)$, $d_{2}^{*}\left(4,1\right)$, Ma(5), Eu(5), and *d*_2_(5) based on the 20 artificial genomes described in the “Materials and Methods” section and the results are given in Table 2. The 20 donor genomes were sorted by the Manhattan distance of the tetra-mer frequencies between the donor and *E. coli* K12.

**Table 2:** Performance of different alignment-free HGT detection methods over 20 artificial genomes with different donor genomes.

**Donor**	**Distance**	***CVT*(3)**	***CVT*(4)**	$d_{2}^{*}\left(3,1\right)$	$d_{2}^{*}\left(4,1\right)$	**Ma(5)**	**Eu(5)**	***d*_2_(5)**
*S. sonnei*	0.027	0.18 ± 0.03	0.18 ± 0.03	0.17 ± 0.03	0.17 ± 0.04	0.16 ± 0.02	0.16 ± 0.02	0.17 ± 0.03
*E. fergusonii*	0.038	0.19 ± 0.02	0.15 ± 0.02	0.19 ± 0.02	0.18 ± 0.02	0.17 ± 0.02	0.16 ± 0.02	0.18 ± 0.02
*E. albertii*	0.044	0.21 ± 0.02	0.17 ± 0.02	0.21 ± 0.01	0.21 ± 0.02	0.17 ± 0.02	0.17 ± 0.02	0.18 ± 0.02
*S. typhimurium*	0.090	0.23 ± 0.02	0.19 ± 0.02	0.23 ± 0.02	0.22 ± 0.02	0.25 ± 0.01	0.27 ± 0.01	0.27 ± 0.01
*E. hermannii*	0.119	0.16 ± 0.01	0.27 ± 0.02	0.14 ± 0.02	0.15 ± 0.02	0.26 ± 0.02	0.26 ± 0.02	0.25 ± 0.02
*P. ananatis*	0.123	0.23 ± 0.02	0.38 ± 0.02	0.19 ± 0.02	0.21 ± 0.03	0.26 ± 0.01	0.26 ± 0.01	0.25 ± 0.02
*B. goodwinii*	0.124	0.27 ± 0.02	0.44 ± 0.02	0.27 ± 0.02	0.29 ± 0.02	0.30 ± 0.02	0.32 ± 0.03	0.32 ± 0.02
*E. cloacae*	0.141	0.23 ± 0.02	0.28 ± 0.02	0.19 ± 0.03	0.21 ± 0.02	0.32 ± 0.02	0.30 ± 0.02	0.30 ± 0.02
*Y. pestis*	0.160	0.51 ± 0.02	0.61 ± 0.02	0.50 ± 0.02	0.56 ± 0.02	0.33 ± 0.03	0.30 ± 0.03	0.37 ± 0.02
*E. vulneris*	0.223	0.39 ± 0.02	0.27 ± 0.02	0.29 ± 0.01	0.33 ± 0.01	0.46 ± 0.02	0.44 ± 0.02	0.43 ± 0.02
*K. pneumoniae*	0.228	0.28 ± 0.03	0.26 ± 0.03	0.21 ± 0.02	0.23 ± 0.01	0.46 ± 0.02	0.46 ± 0.03	0.43 ± 0.01
*V. parahaemolyticus*	0.261	0.87 ± 0.01	0.85 ± 0.01	0.88 ± 0.01	0.88 ± 0.00	0.55 ± 0.02	0.51 ± 0.04	0.58 ± 0.01
*P. luminescens*	0.283	0.60 ± 0.02	0.65 ± 0.03	0.59 ± 0.01	0.63 ± 0.02	0.56 ± 0.01	0.55 ± 0.01	0.57 ± 0.01
*A. ferrooxidans*	0.301	0.71 ± 0.02	0.68 ± 0.02	0.62 ± 0.02	0.63 ± 0.02	0.54 ± 0.01	0.52 ± 0.03	0.52 ± 0.01
*B. abortus*	0.308	0.86 ± 0.01	0.82 ± 0.01	0.85 ± 0.01	0.85 ± 0.01	0.63 ± 0.01	0.60 ± 0.01	0.55 ± 0.01
*L. pneumophila*	0.449	0.84 ± 0.01	0.78 ± 0.01	0.84 ± 0.01	0.87 ± 0.00	0.85 ± 0.01	0.82 ± 0.02	0.84 ± 0.02
*X. axonopodis*	0.487	0.87 ± 0.01	0.86 ± 0.01	0.83 ± 0.01	0.82 ± 0.01	0.85 ± 0.03	0.85 ± 0.03	0.76 ± 0.02
*P. aeruginosa*	0.550	0.89 ± 0.00	0.79 ± 0.01	0.86 ± 0.01	0.81 ± 0.01	0.90 ± 0.01	0.89 ± 0.01	0.81 ± 0.01
*B. pseudomallei*	0.682	0.96 ± 0.01	0.87 ± 0.01	0.95 ± 0.01	0.94 ± 0.02	0.90 ± 0.02	0.90 ± 0.03	0.88 ± 0.03
*C. coli*	0.713	0.97 ± 0.00	0.94 ± 0.01	0.97 ± 0.00	0.98 ± 0.00	0.98 ± 0.00	0.97 ± 0.00	0.97 ± 0.00

*The first column shows the donor genome of the artificial genome. The top 12 species have the same order level as E. coli and the bottom 8 species have different order level from E. coli. The second column is the Manhattan distance between donor genome and E. coli K12 based on tetranucletide frequency. The third to the ninth columns are the optimal F_1_-score of different methods over different artificial genomes. The optimal F_1_ scores for each donor genome are highlighted*.

We divided the donor genomes into three groups separated by horizontal lines in Table 2. For the top group of donor genomes with Manhattan distance between the donor and host genomes less than 0.12, none of the methods have *F*_1_ value greater than 0.30 indicating that none of them can successfully detect HGT when the donor genome and host genome are very close. For the second group of donor genomes with Manhattan distance between 0.12 to 0.31, for eight out of ten donor genomes except for *V. parahaemolyticus* and *B. abortus*, the optimal *F*_1_ scores are moderate between 0.32 to 0.71. Except for *E. cloacae, E. vulneris* and *K. pneumoniae*, the background adjusted dissimilarity measures outperform the non-background adjusted measures, some times by a significant margin. For example, when the donor genome is *V. parahaemolyticus*, the *F*_1_-scores for *CVT*(3), *CVT*(4), $d_{2}^{*}\left(3,1\right)$, and $d_{2}^{*}\left(4,1\right)$ are all at least 0.85, while the *F*_1_-scores for Ma(5), Eu(5), and *d*_2_(5) are at most 0.58. Within this group of donor genomes, *CVT*(4) seems to perform better than *CVT*(3) when the Manhattan distance between the donor and host genomes is between 0.12 and 0.22, while *CVT*(3) is slightly better than *CVT*(4) when the Manhattan distance is between 0.22 to 0.31. The results are reasonable since when the donor and host genomes are relatively close, relative long kmers are needed to separate the transferred fragments from the background. On the other hand, when the donor and host genomes are relatively far apart, relatively short-mers are more discriminative. For the last group of donor genomes with large distances between the donor and host genomes, all the seven methods perform decently well with *CVT*(3), $d_{2}^{*}\left(4,1\right)$ and Ma(5) generally as the best performers.

**Figure 2:**
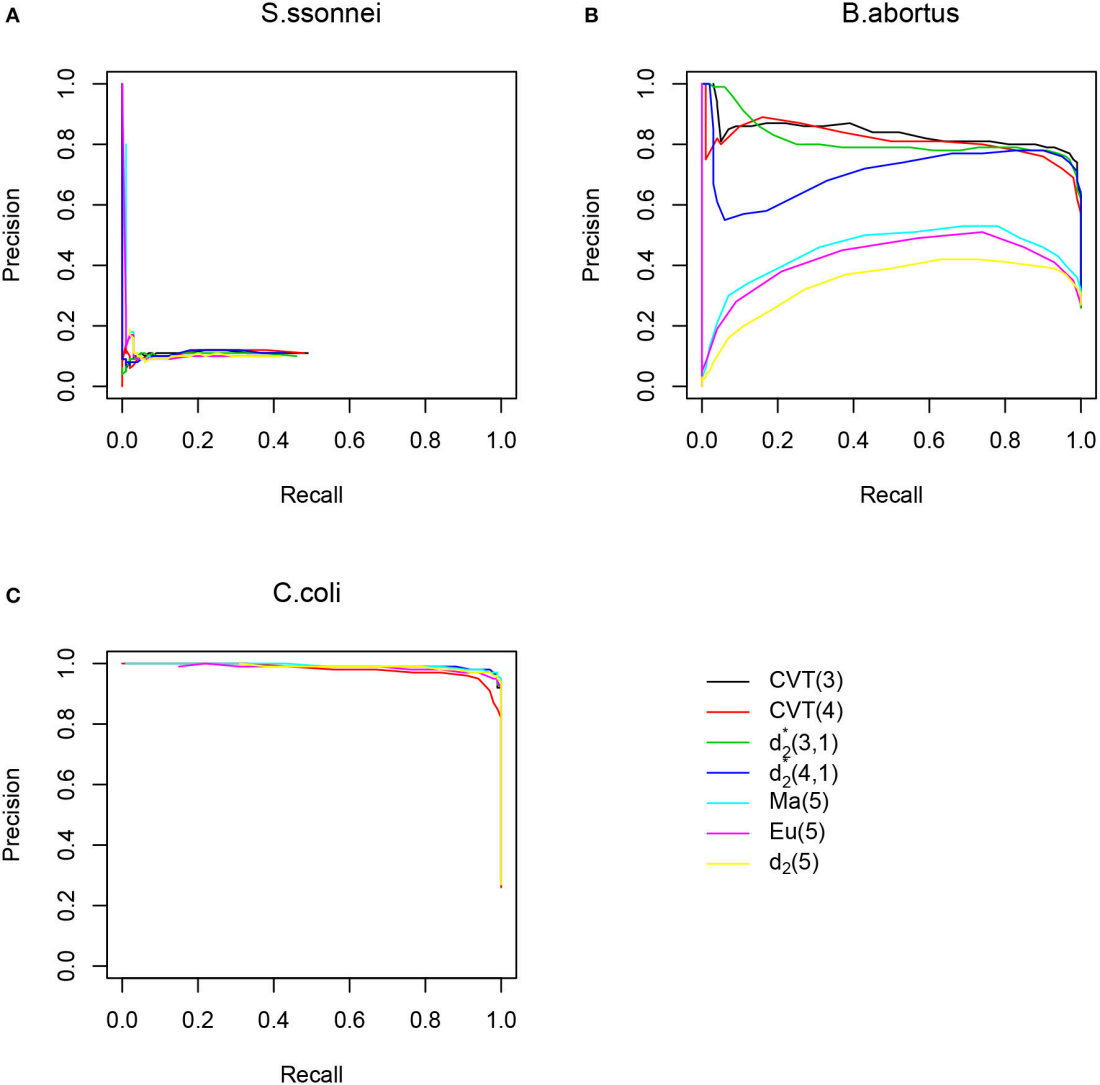
The Precision-Recall Curves (PRC) of different HGT detection methods along artificial genomes using *E. coli* as host genome. **(A)** PRC when using *S.sonnei* as donor genome, no methods performs well. **(B)** PRC when using *B. abortus* as donor genome, *CVT*(3), *CVT*(4),$d_{2}^{*}\left(3,1\right)$ and $d_{2}^{*}\left(4,1\right)$ outperform other methods. **(C)** PRC when using *C.coli* as donor genome, all methods perform reasonably well.

In addition to the comparison of the different methods based on the optimal *F*_1_-score, we also plotted the precision-recall curves for three donor genomes *S. sonnei, B. abortus*, and *C. coli* in Figure 2 as examples for each group. Similar results for the relative performance of the different methods as based on *F*_1_-scores were observed.

### The performance of the alignment-free methods increases with the window size within the range of 3–8 kbp

We further studied the influence of window size on different methods as the performances of alignment-free methods always reply on the sequence length that should be long enough to represent the genomic signature. Besides 5 kbp window size with 500 bp sliding step, we also checked the performance of different methods based on 3 kbp window size with 300 bp sliding step and 8kbp window size with 800 bp sliding step by using the same evaluation approach. Among the 20 artificial genomes that have been generated to study the influence of the genetic distance between the donor genome and host genome, we chose 8 of them in which donors have different order level from that of *E. coli* K12. Optimal *F*_1_ score of different methods using different window sizes over these 8 genomes are shown in Table 3. All methods showed similar trend that their mean *F*_1_ score increases as the window length increases from 3 to 8 kbp. But *CVT*(3) is the most robust with different window sizes and its performance suffers less with the decrease of window size compared with other methods.

**Table 3:** Performance of different methods over artificial genomes by using different window sizes.

**Donor**	WS^*^ (kbp)	***CVT*(3)**	***CVT*(4)**	$d_{2}^{*}\left(3,1\right)$	$d_{2}^{*}\left(4,1\right)$	**Ma(5)**	**Eu(5)**	***d*_2_(5)**
*V. parahaemolyticus*	3	0.84 ± 0.01	0.82 ± 0.01	0.85 ± 0.01	0.87 ± 0.01	0.47 ± 0.02	0.43 ± 0.02	0.53 ± 0.02
*V. parahaemolyticus*	5	0.87 ± 0.01	0.85 ± 0.01	0.88 ± 0.01	0.88 ± 0.00	0.55 ± 0.02	0.51 ± 0.04	0.58 ± 0.01
*V. parahaemolyticus*	8	0.95 ± 0.01	0.91 ± 0.01	0.95 ± 0.01	0.94 ± 0.01	0.62 ± 0.02	0.60 ± 0.02	0.64 ± 0.01
*A. ferrooxidans*	3	0.65 ± 0.02	0.62 ± 0.01	0.58 ± 0.02	0.59 ± 0.02	0.51 ± 0.02	0.47 ± 0.02	0.49 ± 0.02
*A. ferrooxidans*	5	0.71 ± 0.02	0.68 ± 0.02	0.62 ± 0.02	0.63 ± 0.02	0.54 ± 0.01	0.52 ± 0.03	0.52 ± 0.01
*A. ferrooxidans*	8	0.82 ± 0.03	0.72 ± 0.02	0.68 ± 0.04	0.66 ± 0.02	0.61 ± 0.03	0.56 ± 0.03	0.56 ± 0.02
*B. abortus*	3	0.79 ± 0.01	0.77 ± 0.02	0.78 ± 0.01	0.78 ± 0.01	0.55 ± 0.01	0.52 ± 0.01	0.50 ± 0.01
*B. abortus*	5	0.86 ± 0.01	0.82 ± 0.01	0.85 ± 0.01	0.85 ± 0.01	0.63 ± 0.01	0.60 ± 0.01	0.55 ± 0.01
*B. abortus*	8	0.94 ± 0.01	0.88 ± 0.02	0.93 ± 0.01	0.91 ± 0.01	0.68 ± 0.01	0.66 ± 0.02	0.61 ± 0.02
*L. pneumophila*	3	0.79 ± 0.02	0.73 ± 0.01	0.79 ± 0.01	0.84 ± 0.01	0.79 ± 0.01	0.77 ± 0.01	0.79 ± 0.01
*L. pneumophila*	5	0.84 ± 0.01	0.78 ± 0.01	0.84 ± 0.01	0.87 ± 0.00	0.85 ± 0.01	0.82 ± 0.02	0.84 ± 0.02
*L. pneumophila*	8	0.91 ± 0.02	0.82 ± 0.01	0.89 ± 0.01	0.93 ± 0.01	0.88 ± 0.02	0.86 ± 0.01	0.87 ± 0.01
*X. axonopodis*	3	0.81 ± 0.01	0.81 ± 0.02	0.74 ± 0.01	0.74 ± 0.01	0.78 ± 0.02	0.78 ± 0.03	0.69 ± 0.02
*X. axonopodis*	5	0.87 ± 0.01	0.86 ± 0.01	0.83 ± 0.01	0.82 ± 0.01	0.85 ± 0.03	0.85 ± 0.03	0.76 ± 0.02
*X. axonopodis*	8	0.96 ± 0.01	0.92 ± 0.01	0.92 ± 0.01	0.91 ± 0.02	0.86 ± 0.03	0.82 ± 0.02	0.81 ± 0.02
*P. aeruginosa*	3	0.86 ± 0.01	0.73 ± 0.02	0.79 ± 0.01	0.72 ± 0.01	0.84 ± 0.01	0.83 ± 0.02	0.76 ± 0.01
*P. aeruginosa*	5	0.89 ± 0.00	0.79 ± 0.01	0.86 ± 0.01	0.81 ± 0.01	0.90 ± 0.01	0.89 ± 0.01	0.81 ± 0.01
*P. aeruginosa*	8	0.96 ± 0.01	0.84 ± 0.01	0.95 ± 0.01	0.90 ± 0.01	0.90 ± 0.01	0.88 ± 0.03	0.86 ± 0.01
*B. pseudomallei*	3	0.92 ± 0.01	0.83 ± 0.01	0.91 ± 0.01	0.90 ± 0.01	0.90 ± 0.03	0.91 ± 0.02	0.84 ± 0.03
*B. pseudomallei*	5	0.96 ± 0.01	0.87 ± 0.01	0.95 ± 0.01	0.94 ± 0.02	0.90 ± 0.02	0.90 ± 0.03	0.88 ± 0.03
*B. pseudomallei*	8	0.97 ± 0.01	0.93 ± 0.01	0.96 ± 0.01	0.96 ± 0.01	0.89 ± 0.02	0.89 ± 0.03	0.86 ± 0.02
*C. coli*	3	0.96 ± 0.01	0.90 ± 0.00	0.97 ± 0.01	0.97 ± 0.01	0.97 ± 0.00	0.95 ± 0.01	0.96 ± 0.00
*C. coli*	5	0.97 ± 0.00	0.94 ± 0.01	0.97 ± 0.00	0.98 ± 0.00	0.98 ± 0.00	0.97 ± 0.00	0.97 ± 0.00
*C. coli*	8	0.93 ± 0.01	0.96 ± 0.01	0.94 ± 0.00	0.96 ± 0.01	0.97 ± 0.00	0.96 ± 0.01	0.96 ± 0.00

*Values in the second column are the window sizes. All the other columns are the same as in Table 2. The optimal F_1_ scores for each donor genome by using different window sizes are highlighted*.

* *WS, window size*.

### Robustness of the relative performance of the different methods with respect to different host genomes

To see the robustness of our results on the relative performance of the different alignment-free HGT detection methods with respect to host genomes, we changed the host genome from *E. coli* to *B. abortus* and *K. pneumoniae*, respectively. The complete results are given as Tables S2, S3 in Supplementary Material. From both tables, it can be seen that the conclusions about the relative performance of the different methods hold regardless of the host genome.

### Applications to real HGT data support the good performance of background adjusted dissimilarity measures

#### Evaluation of different methods based on 118 genomes with known HGT genomic islands

**Figure 3:**
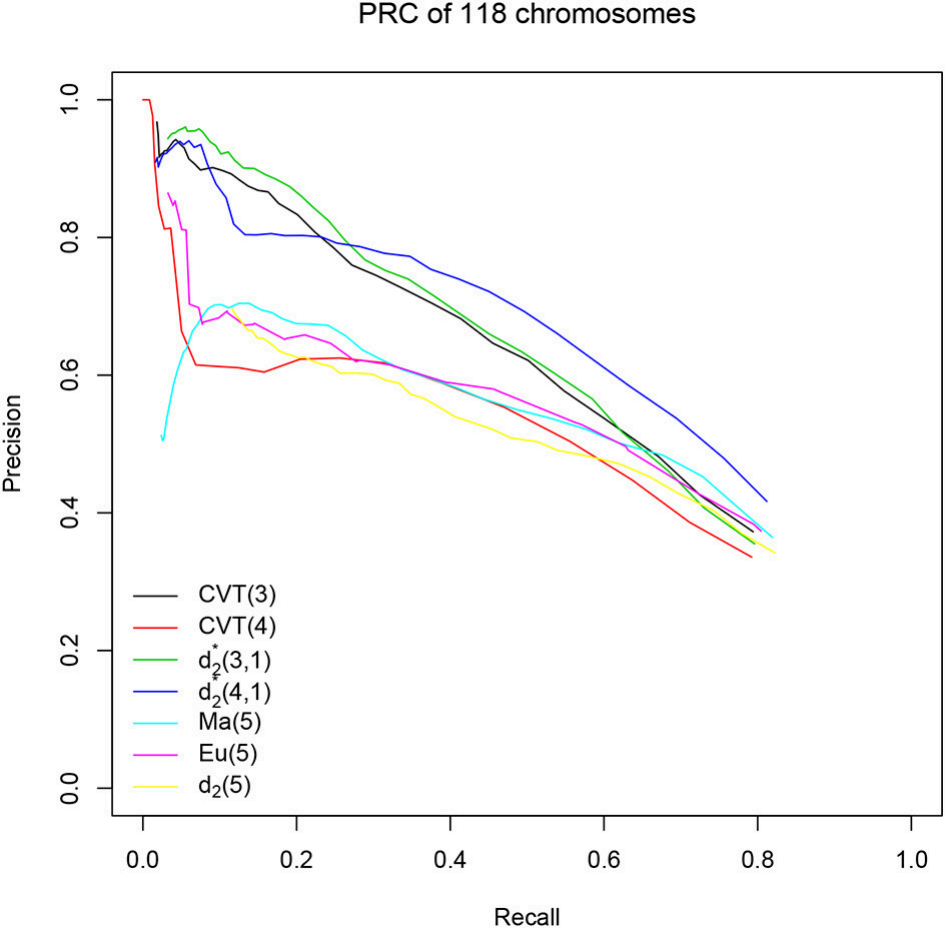
The Precision-Recall Curves (PRC) of the different methods based on 118 genomes with known HGT genomic islands.

We next applied the various dissimilarity measures to identify genomic islands generated from HGT for the 118 genomes described in the “Materials and Methods" section. We still chose 40 thresholds as in our simulation studies for each method, and calculated the optimal accuracy that is the highest accuracy one method can achieve under certain threshold. The results are shown in part (a) of Table 4. The values of the optimal accuracy for the different methods are not markedly different, but we can still see that the background adjusted dissimilarity measures *CVT*(3), *CVT*(4), $d_{2}^{*}\left(3,1\right)$, and $d_{2}^{*}\left(4,1\right)$ have slightly higher accuracy than the non-background adjusted dissimilarity measures *Eu*(5), *Ma*(5), and *d*_2_(5). Similarly, we also evaluated the different methods based on the optimal *F*_1_-score as shown in part (b) of Table 4. The conclusions on the relative performance of the methods based on *F*_1_-score are essentially the same as that based on optimal accuracy. In addition, we also plotted the precision-recall curves of the different methods based on this data set and the resulting figures are shown in Figure 3. It is clear from the figure that *CVT*(3), $d_{2}^{*}\left(3,1\right)$ and $d_{2}^{*}\left(4,1\right)$ perform much better than the other methods. In Langille et al. (2008), SIGI-HMM and IslandPath/DIMOB showed the highest accuracy of 0.86. We did not include them in our comparison because they incorporate other information such as codon uasge, dinucleotide bias, gene expression and mobility that can only be used when the genome is annotated. However, in terms of accuracy, $d_{2}^{*}\left(4,1\right)$ can achieve the same performance as SIGI-HMM and IslandPath/DIMOB by detecting HGT purely based on the genomic composition.

**Table 4:** Performance of different methods over 118 genomes with known HGT genomic islands in Langille et al. (2008) based on (a) optimal accuracy and (b) optimal *F*_1_-score.

**Method**	**(a) Based on accuracy**			**(b) Based on** ***F***_**1**_ **score**		
	**Precision**	**Recall**	**Optimal accuracy**	**Precision**	**Recall**	**Optimal *F*_1_ score**
*CVT*(3)	0.68	0.41	0.84	0.54	0.60	0.57
*CVT*(4)	0.62	0.31	0.83	0.50	0.56	0.53
$d_{2}^{*}$(3, 1)	0.72	0.38	0.85	0.57	0.58	0.58
$d_{2}^{*}$(4, 1)	0.72	0.45	0.86	0.58	0.63	0.61
Ma(5)	0.67	0.26	0.83	0.48	0.68	0.56
Eu(5)	0.58	0.46	0.83	0.50	0.63	0.55
*d*_2_(5)	0.60	0.30	0.82	0.45	0.67	0.53

*The second and third columns show the precision and recall to achieve the optimal accuracy given in the fourth column. The fifth and sixth columns show the precision and recall corresponding to the optimal F_1_-score given in the seventh column*.

#### Evaluation of different methods based on *E. faecalis V583* with known seven HGT genes

In *E. faecalis V583*, a genomic region that contains 7 genes (EF2293-EF2299) conferring vancomycin resistance to *E. faecalis* has been known to have been horizontally transferred (Tsirigos and Rigoutsos, 2005). In this case, we calculated the distance between each gene and the *E. faecalis V583* genome using different methods. We then ranked all 3112 *E. faecalis V583* genes by the distance in descending order where the first gene has the largest distance to *E. faecalis V583* genome. Better HGT detection methods should give EF2293-EF2299 lower ranks. Ranks of EF2293-EF2299 and the median and mean rank of these 7 genes for all the methods are shown in Table 5. $d_{2}^{*}\left(3,1\right)$ gives lower median and mean ranks for EF2293-EF2299 than other methods. In comparison with $d_{2}^{*}$, the median and mean ranks given by more commonly-used *Manhanttan* and *Euclidean* distances are larger than 1,000, which are unreasonably high considering the fact that the HGT proportions in most bacteria genomes range from only 2 to 15% (Garcia-Vallvé et al., 2000).

**Table 5:** The distances between each gene and *E. faecalis V583* genome were calculated and genes were ranked by their distances.

**Gene**	***CVT*(3)**	***CVT*(4)**	$d_{2}^{*}\left(3,1\right)$	$d_{2}^{*}\left(4,1\right)$	**Ma(5)**	**Eu(5)**	***d*_2_(5)**
EF2293	607	815	688	605	854	1,001	511
EF2294	325	1,874	222	447	1,302	1,373	719
EF2295	138	855	109	219	1,169	1,273	520
EF2296	379	1,613	313	385	1,392	1,491	850
EF2297	618	2,638	665	1,245	1,117	1,165	551
EF2298	660	1,355	702	772	1,978	1,924	1,025
EF2299	687	1,084	477	607	814	820	384
Median	607	1,355	477	605	1,169	1,273	551
Mean	487.7	1,462.0	453.7	611.4	1,232.3	1,292.4	651.4

*The first to seventh rows show the ranks of EF2293-EF2299 among all E.faecalis V583 genes calculated by different methods. The eighth and ninth rows show the median and mean of the ranks of the seven genes*.

## Discussion

Kmer-based alignment-free methods have been used to detect horizontal gene transfers in bacterial genomes (Dufraigne et al., 2005; Tsirigos and Rigoutsos, 2005; Rajan et al., 2007). There are a number of advantages of kmer-based methods over other alignment-free methods or alignment-based methods. First of all, kmer-based methods are time efficient and memory friendly by avoiding alignment and topological data analysis. Secondly, kmer-based methods do not rely on phylogenetic relationships among multiple organisms, which enables them to detect HGTs from a single unannotated genome. In addition, kmer-based methods are able to detect HGTs in both coding and non-coding regions.

In this study, we investigated the potential of using recently developed alignment-free sequence comparison statistics, in particular, *CVTree*, $d_{2}^{*}$ and $d_{2}^{S}$, that adjust for the background word frequencies, for horizontal gene transfer detection. Although many composition based methods have been used for HGT detection, to the best of our knowledge, the background adjusted statistics have not been used for HGT detection.

We first generated simulated artificial genomes with HGT by using *E. coli* K12 as the host genome and inserted sequences uniformly chosen from other genomes into it. We then evaluated the performance of kmer-based alignment-free methods of different distance measures, kmer length and Markov order on HGT detection of artificial genomes. Based on the results, we reduced our set to *CVTree*(*k* = 3), *CVTree*(*k* = 4), $d_{2}^{*}\left(k\;=\;3,m\;=\;1\right)$, $d_{2}^{*}\left(k=4,m=1\right)$, Ma(*k* = 5), Eu(*k* = 5), and *d*_2_(*k* = 5) for more detailed comparisons including influence of different factors and their performance on real data sets.

As a conclusion, we evaluated the performance of kmer-based alignment-free methods with different dissimilarity measures, kmer length and Markov order on both artificial genomes and real data sets. Our results suggest the background adjusted dissimilarity measures, *CVTree*, $d_{2}^{*}$ and $d_{2}^{S}$, generally perform better than the non-background adjusted measures based on Euclidean and Manhattan distances or *d*_2_. In terms of word length, *k* = 3 or *k* = 4 seems to perform well in both our simulation and real data analysis.

Although kmer-based alignment-free methods for HGT detection are more time and memory efficient than alignment-based methods and they do not depend on genome annotation or evolutionary tree, they also have limits. First of all, their performances depend on the evolutionary distance between host and donor genomes. Our study showed alignment-free methods are suitable for HGT detection when host and donor genomes are in different order levels. In addition, the size of sliding window is the smallest length of HGT that can be detected by the kmer-based alignment-free methods, so they are not suitable for identifying HGT smaller than 5 kbp. Furthermore, they are not likely to detect HGT that occurred in the very distant past, as these sequences transferred from the donor genome will ameliorate to reflect the DNA composition of the host genome over time (Lawrence and Ochman, 1997). Finally, the detected atypical regions could be explained by some other reasons. For example, rRNA regions can have their own genomic signatures (Nicolas et al., 2002; Dufraigne et al., 2005), which differ from the host signature, but this does not imply that they are horizontally transferred.

Therefore, alignment-free methods are not aimed to replace alignment-based methods in all cases. Instead, they are complementary as each has unique advantages in different scenarios and they also tend to find complementary sets of HGT regions (Tamames and Moya, 2008). Alignment-free methods are preferred when no evolutionary trees are available or genomes are not annotated, which is common in many studies. The findings of our study suggest *CVTree* with word length of 3, $d_{2}^{*}$ with word length 3, Markov order 1 and $d_{2}^{*}$ with word length 4, Markov order 1 perform well in most situations.

## Author contributions

KT implemented and carried out the computational analyses and wrote the paper. YL provided software for calculating alignment-free dissimilarity measures. FS led the project and finalized the paper. All authors agree to the content of the final paper.

### Conflict of interest statement

The authors declare that the research was conducted in the absence of any commercial or financial relationships that could be construed as a potential conflict of interest.
