# Supplementary material for: Background Adjusted Alignment-Free Dissimilarity Measures Improve the Detection of Horizontal Gene Transfer
Source: Front Microbiol. 2018 Apr 16;9:711. doi: 10.3389/fmicb.2018.00711 (PMC5911508; doi:10.3389/fmicb.2018.00711)
Supplement: Supplementary file 1 [file Table1.pdf]

Background Adjusted Alignment-free Dissimilarity Measures Improve the Detection of Horizontal Gene Transfer: Supplementary Material

Kujin Tang[1], Yang Young Lu[1], and Fengzhu Sun *[1,2]

[1]Molecular and Computational Biology Program, Department of Biological Sciences, University of Southern California, CA, USA
[2]Centre for Computational Systems Biology, School of Mathematical Sciences, Fudan University, Shanghai, China

*To whom correspondence should be addressed. Tel: +1 (213)-740-2413; Fax: +1 (213)-740-8631; Email: fsun@usc.edu

Host	Start	End	Donor	Start	End	Length
E. coli	345278	380961	*M. tuberculosis*	3905301	3940984	35684
E. coli	388702	405082	*S. pneumoniae*	1899095	1915475	16381
E. coli	523074	536488	*H. influenzae*	1570632	1584046	13415
E. coli	609097	644659	*M. tuberculosis*	56261	91823	35563
E. coli	880226	906410	*S. pneumoniae*	69014	95198	26185
E. coli	1102764	1114083	*M. tuberculosis*	1354709	1366028	11320
E. coli	1314037	1352941	*H. pylori*	125023	163927	38905
E. coli	1363730	1378984	*H. pylori*	499545	514799	15255
E. coli	1412972	1442804	*M. tuberculosis*	2659436	2689268	29833
E. coli	1563095	1595311	*B. subtilis*	2750746	2782962	32217
E. coli	2133106	2153111	*B. subtilis*	2472809	2492814	20006
E. coli	2418973	2431888	*M. tuberculosis*	3267094	3280009	12916
E. coli	2661049	2686710	*B. subtilis*	2471084	2496745	25662
E. coli	2739292	2762176	*S. pneumoniae*	3745	26629	22885
E. coli	2964060	2975795	*M. tuberculosis*	2700695	2712430	11736
E. coli	3049010	3058543	*B. subtilis*	2069861	2079394	9534
E. coli	3196491	3216327	*B. subtilis*	3287257	3307093	19837
E. coli	3865131	3873654	*H. pylori*	299584	308107	8524
E. coli	4148122	4188039	*H. influenzae*	1208124	1248041	39918
E. coli	4260138	4279066	*H. influenzae*	435547	454475	18929
E. coli	4536358	4549293	*S. pneumoniae*	1261210	1274145	12936
E. coli	4909481	4923174	*H. influenzae*	1336445	1350138	13694

Table S1: Detailed composition of one of the 10 *E. coli*_artificial genomes. The second and third columns are the start and end positions of this insertion in the *E. coli*_artificial genome. The fifth and sixth columns are the position of this fragment in the donor genoms. The seventh column is the length of the transferred fragment.

Donor	Distance	$CVT(3)$	$CVT(4)$	$d_2^*(3,1)$	$d_2^*(4,1)$	Ma(5)	Eu(5)	$d_2(5)$
A. ferrooxidans	0.205	$0.71^{\pm 0.01}$	$0.36^{\pm 0.02}$	$0.70^{\pm 0.01}$	$0.68^{\pm 0.01}$	$0.68^{\pm 0.01}$	$0.63^{\pm 0.03}$	$0.66^{\pm 0.01}$
K. pneumoniae	0.223	$0.74^{\pm 0.01}$	$0.46^{\pm 0.02}$	$0.72^{\pm 0.02}$	$0.69^{\pm 0.01}$	$0.72^{\pm 0.01}$	$0.68^{\pm 0.01}$	$0.69^{\pm 0.01}$
B. goodwinii	0.237	$0.76^{\pm 0.01}$	$0.57^{\pm 0.03}$	$0.74^{\pm 0.01}$	$0.70^{\pm 0.01}$	$0.71^{\pm 0.01}$	$0.67^{\pm 0.01}$	$0.68^{\pm 0.01}$
E. hermannii	0.245	$0.77^{\pm 0.01}$	$0.51^{\pm 0.02}$	$0.76^{\pm 0.01}$	$0.72^{\pm 0.01}$	$0.71^{\pm 0.01}$	$0.68^{\pm 0.01}$	$0.69^{\pm 0.01}$
E. cloacae	0.258	$0.77^{\pm 0.01}$	$0.47^{\pm 0.02}$	$0.77^{\pm 0.01}$	$0.73^{\pm 0.01}$	$0.75^{\pm 0.01}$	$0.71^{\pm 0.01}$	$0.74^{\pm 0.01}$
E. vulneris	0.258	$0.81^{\pm 0.01}$	$0.47^{\pm 0.02}$	$0.77^{\pm 0.01}$	$0.73^{\pm 0.01}$	$0.75^{\pm 0.00}$	$0.72^{\pm 0.02}$	$0.73^{\pm 0.01}$
P. ananatis	0.271	$0.79^{\pm 0.01}$	$0.56^{\pm 0.04}$	$0.77^{\pm 0.01}$	$0.73^{\pm 0.01}$	$0.75^{\pm 0.01}$	$0.71^{\pm 0.01}$	$0.74^{\pm 0.02}$
S. typhimurium	0.273	$0.79^{\pm 0.01}$	$0.55^{\pm 0.02}$	$0.78^{\pm 0.01}$	$0.73^{\pm 0.01}$	$0.75^{\pm 0.01}$	$0.71^{\pm 0.01}$	$0.73^{\pm 0.02}$
E. coli	0.308	$0.80^{\pm 0.01}$	$0.51^{\pm 0.04}$	$0.78^{\pm 0.02}$	$0.73^{\pm 0.01}$	$0.77^{\pm 0.02}$	$0.74^{\pm 0.02}$	$0.76^{\pm 0.01}$
S. sonnei	0.315	$0.82^{\pm 0.02}$	$0.54^{\pm 0.02}$	$0.78^{\pm 0.01}$	$0.74^{\pm 0.01}$	$0.77^{\pm 0.01}$	$0.74^{\pm 0.01}$	$0.77^{\pm 0.01}$
X. axonopodis	0.324	$0.78^{\pm 0.02}$	$0.54^{\pm 0.03}$	$0.71^{\pm 0.02}$	$0.67^{\pm 0.01}$	$0.75^{\pm 0.02}$	$0.75^{\pm 0.02}$	$0.68^{\pm 0.02}$
E. albertii	0.332	$0.81^{\pm 0.02}$	$0.55^{\pm 0.02}$	$0.78^{\pm 0.01}$	$0.74^{\pm 0.01}$	$0.78^{\pm 0.01}$	$0.75^{\pm 0.01}$	$0.77^{\pm 0.01}$
E. fergusonii	0.334	$0.82^{\pm 0.02}$	$0.50^{\pm 0.01}$	$0.79^{\pm 0.01}$	$0.74^{\pm 0.01}$	$0.78^{\pm 0.00}$	$0.75^{\pm 0.01}$	$0.77^{\pm 0.01}$
P. aeruginosa	0.392	$0.78^{\pm 0.02}$	$0.47^{\pm 0.03}$	$0.68^{\pm 0.02}$	$0.63^{\pm 0.01}$	$0.83^{\pm 0.01}$	$0.83^{\pm 0.02}$	$0.75^{\pm 0.01}$
Y. pestis	0.414	$0.87^{\pm 0.01}$	$0.70^{\pm 0.03}$	$0.85^{\pm 0.01}$	$0.83^{\pm 0.01}$	$0.86^{\pm 0.02}$	$0.85^{\pm 0.01}$	$0.85^{\pm 0.02}$
V. parahaemolyticus	0.475	$0.90^{\pm 0.01}$	$0.84^{\pm 0.01}$	$0.90^{\pm 0.01}$	$0.90^{\pm 0.01}$	$0.94^{\pm 0.01}$	$0.94^{\pm 0.01}$	$0.94^{\pm 0.02}$
B. pseudomallei	0.502	$0.84^{\pm 0.01}$	$0.52^{\pm 0.02}$	$0.82^{\pm 0.02}$	$0.77^{\pm 0.01}$	$0.88^{\pm 0.02}$	$0.90^{\pm 0.03}$	$0.79^{\pm 0.02}$
P. luminescens	0.531	$0.86^{\pm 0.02}$	$0.70^{\pm 0.02}$	$0.84^{\pm 0.01}$	$0.83^{\pm 0.01}$	$0.92^{\pm 0.02}$	$0.91^{\pm 0.02}$	$0.91^{\pm 0.02}$
L. pneumophila	0.646	$0.92^{\pm 0.01}$	$0.73^{\pm 0.01}$	$0.93^{\pm 0.01}$	$0.92^{\pm 0.01}$	$0.97^{\pm 0.01}$	$0.95^{\pm 0.02}$	$0.93^{\pm 0.00}$
C. coli	0.877	$0.97^{\pm 0.01}$	$0.87^{\pm 0.01}$	$0.95^{\pm 0.01}$	$0.95^{\pm 0.01}$	$0.97^{\pm 0.00}$	$0.96^{\pm 0.00}$	$0.90^{\pm 0.01}$

Table S2: Performances of different methods over artificial genomes with *B. abortus* as host genome and different donor genomes. Values in 2nd column are the Manhattan distance between donor genome and *B. abortus* based on tetranucletide frequency. The third to the ninth columns are the optimal F_1-score of different methods over different artificial genomes.

Donor	Distance	$CVT(3)$	$CVT(4)$	$d_2^*(3,1)$	$d_2^*(4,1)$	Ma(5)	Eu(5)	$d_2(5)$
E. vulneris	0.174	$0.33^{\pm0.01}$	$0.18^{\pm0.01}$	$0.33^{\pm0.02}$	$0.31^{\pm0.02}$	$0.16^{\pm0.02}$	$0.14^{\pm0.02}$	$0.20^{\pm0.02}$
E. cloacae	0.223	$0.23^{\pm0.01}$	$0.29^{\pm0.01}$	$0.26^{\pm0.01}$	$0.30^{\pm0.01}$	$0.17^{\pm0.01}$	$0.16^{\pm0.01}$	$0.25^{\pm0.01}$
E. hermannii	0.541	$0.19^{\pm0.01}$	$0.36^{\pm0.01}$	$0.20^{\pm0.01}$	$0.27^{\pm0.01}$	$0.16^{\pm0.01}$	$0.13^{\pm0.02}$	$0.23^{\pm0.01}$
P. ananatis	0.880	$0.22^{\pm0.01}$	$0.45^{\pm0.01}$	$0.22^{\pm0.01}$	$0.32^{\pm0.02}$	$0.18^{\pm0.02}$	$0.16^{\pm0.02}$	$0.28^{\pm0.02}$
B. goodwinii	0.121	$0.32^{\pm0.02}$	$0.43^{\pm0.02}$	$0.32^{\pm0.02}$	$0.36^{\pm0.02}$	$0.22^{\pm0.02}$	$0.20^{\pm0.02}$	$0.29^{\pm0.02}$
A. ferrooxidans	0.228	$0.64^{\pm0.01}$	$0.52^{\pm0.01}$	$0.60^{\pm0.01}$	$0.58^{\pm0.01}$	$0.32^{\pm0.01}$	$0.26^{\pm0.02}$	$0.37^{\pm0.01}$
S. typhimurium	0.256	$0.26^{\pm0.02}$	$0.31^{\pm0.01}$	$0.27^{\pm0.02}$	$0.33^{\pm0.02}$	$0.24^{\pm0.02}$	$0.21^{\pm0.02}$	$0.31^{\pm0.02}$
B. abortus	0.140	$0.76^{\pm0.01}$	$0.73^{\pm0.02}$	$0.74^{\pm0.01}$	$0.74^{\pm0.01}$	$0.41^{\pm0.01}$	$0.28^{\pm0.07}$	$0.44^{\pm0.01}$
E. coli	0.087	$0.30^{\pm0.01}$	$0.32^{\pm0.02}$	$0.31^{\pm0.01}$	$0.38^{\pm0.01}$	$0.30^{\pm0.02}$	$0.27^{\pm0.01}$	$0.40^{\pm0.01}$
S. sonnei	0.624	$0.30^{\pm0.02}$	$0.34^{\pm0.01}$	$0.32^{\pm0.02}$	$0.39^{\pm0.02}$	$0.30^{\pm0.02}$	$0.27^{\pm0.02}$	$0.41^{\pm0.02}$
E. fergusonii	0.348	$0.34^{\pm0.02}$	$0.33^{\pm0.01}$	$0.35^{\pm0.01}$	$0.41^{\pm0.01}$	$0.35^{\pm0.02}$	$0.32^{\pm0.03}$	$0.46^{\pm0.02}$
E. albertii	0.183	$0.37^{\pm0.02}$	$0.34^{\pm0.02}$	$0.37^{\pm0.02}$	$0.43^{\pm0.02}$	$0.34^{\pm0.01}$	$0.31^{\pm0.01}$	$0.45^{\pm0.02}$
X. axonopodis	0.231	$0.82^{\pm0.01}$	$0.68^{\pm0.01}$	$0.75^{\pm0.01}$	$0.69^{\pm0.01}$	$0.58^{\pm0.02}$	$0.53^{\pm0.03}$	$0.47^{\pm0.01}$
Y. pestis	0.447	$0.55^{\pm0.01}$	$0.61^{\pm0.01}$	$0.54^{\pm0.01}$	$0.59^{\pm0.01}$	$0.55^{\pm0.02}$	$0.49^{\pm0.02}$	$0.61^{\pm0.02}$
P. aeruginosa	0.316	$0.84^{\pm0.01}$	$0.56^{\pm0.01}$	$0.79^{\pm0.01}$	$0.67^{\pm0.01}$	$0.60^{\pm0.02}$	$0.56^{\pm0.01}$	$0.47^{\pm0.00}$
V. parahaemolyticus	0.260	$0.84^{\pm0.01}$	$0.84^{\pm0.01}$	$0.86^{\pm0.01}$	$0.86^{\pm0.01}$	$0.79^{\pm0.01}$	$0.76^{\pm0.01}$	$0.79^{\pm0.01}$
P. luminescens	0.340	$0.62^{\pm0.01}$	$0.63^{\pm0.01}$	$0.60^{\pm0.01}$	$0.64^{\pm0.01}$	$0.74^{\pm0.03}$	$0.73^{\pm0.03}$	$0.75^{\pm0.03}$
B. pseudomallei	0.476	$0.92^{\pm0.01}$	$0.69^{\pm0.01}$	$0.89^{\pm0.01}$	$0.87^{\pm0.01}$	$0.80^{\pm0.02}$	$0.85^{\pm0.02}$	$0.65^{\pm0.02}$
L. pneumophila	0.161	$0.79^{\pm0.01}$	$0.74^{\pm0.02}$	$0.78^{\pm0.01}$	$0.82^{\pm0.01}$	$0.93^{\pm0.01}$	$0.91^{\pm0.03}$	$0.93^{\pm0.01}$
C. coli	0.168	$0.97^{\pm0.00}$	$0.90^{\pm0.01}$	$0.97^{\pm0.01}$	$0.96^{\pm0.01}$	$0.97^{\pm0.00}$	$0.96^{\pm0.00}$	$0.97^{\pm0.00}$

Table S3: Performances of different methods over artificial genomes with *K. pneumoniae* as host genome and different donor genomes. Values in 2nd column are the Manhattan distance between donor genome and *K. pneumoniae* based on tetranucletide frequency. The third to the ninth columns are the optimal F_1-score of different methods over different artificial genomes.
